# CD105 is a prognostic marker and valid endothelial target for microbubble platforms in cholangiocarcinoma

**DOI:** 10.1007/s13402-020-00530-8

**Published:** 2020-05-28

**Authors:** Amit Nair, Nicola Ingram, Eldo T. Verghese, Imeshi Wijetunga, Alexander F. Markham, Judy Wyatt, K. Rajendra Prasad, P. Louise Coletta

**Affiliations:** 1 Leeds Institute of Medical Research, St James’s University Hospital, Leeds, LS9 7TF UK; 2 Department of Histopathology, St James’s University Hospital, Leeds, LS9 7TF UK; 3 Department of Hepatobiliary and Transplant Surgery, St James’s University Hospital, Leeds, LS9 7TF UK

**Keywords:** Perihilar cholangiocarcinoma, Bile duct cancer, Biliary tract cancer, Endoglin, CD105, Microbubble

## Abstract

**Purpose:**

The current treatment outcomes in cholangiocarcinoma are poor with cure afforded only by surgical extirpation. The efficacy of targeting the tumoural endothelial marker CD105 in cholangiocarcinoma, as a basis for potential microbubble-based treatment, is unknown and was explored here.

**Methods:**

Tissue expression of CD105 was quantified using immunohistochemistry in 54 perihilar cholangiocarcinoma samples from patients who underwent resection in a single centre over a ten-year period, and analysed against clinicopathological data. In vitro flow assays using microbubbles functionalised with CD105 antibody were conducted to ascertain specificity of binding to murine SVR endothelial cells. Finally, CD105-microbubbles were intravenously administered to 10 Balb/c nude mice bearing heterotopic subcutaneous human extrahepatic cholangiocarcinoma (TFK-1 and EGI-1) xenografts after which in vivo binding was assessed following contrast-enhanced destruction replenishment ultrasound application.

**Results:**

Though not significantly associated with any examined clinicopathological variable, we found that higher CD105 expression was independently associated with poorer patient survival (median 12 vs 31 months; *p* = 0.002). In vitro studies revealed significant binding of CD105-microbubbles to SVR endothelial cells in comparison to isotype control (*p* = 0.01), as well as in vivo to TFK-1 (*p* = 0.02) and EGI-1 (*p* = 0.04) mouse xenograft vasculature.

**Conclusion:**

Our results indicate that CD105 is a biomarker eminently suitable for cholangiocarcinoma targeting using functionalised microbubbles.

## Introduction

Cholangiocarcinoma (CCA) is an aggressive malignancy of biliary tract epithelium accounting for 10–15% of primary liver cancers [1]. Peri-hilar cholangiocarcinoma (pCCA) is the most common variant (60–70%) of CCA and is typically associated with locally advanced disease at presentation with widely varying surgical extirpation rates ranging from 28 to 95% [2]. Complete resection with clear (R0) margins nonetheless remains the only means for cure. In addition, the efficacy of chemotherapy in adjuvant or palliative settings is modest [3, 4]. Therefore, new strategies are needed to facilitate earlier pCCA diagnosis and improve its prognosis. Molecular targeting of tumour vasculature is a relatively new therapeutic approach intended to curtail tumour angiogenesis, growth and metastatic potential. Several anti-angiogenic agents are now available for the treatment of certain solid cancers, most of which inhibit the Vascular Endothelial Growth Factor (VEGF) signalling pathway [5]. However, lack of significant therapeutic gain indicates that tumour angiogenesis may be mediated not only through VEGF, but also through several alternative routes [6, 7]. One such signalling pathway involves Transforming Growth Factor (TGF)-β. The pro-angiogenic effects of TGF-β within tumour tissues are mediated via the TGF-β co-receptor CD105 (Endoglin), which is present in abundance within endothelial cells of tumour vasculature [8]. The role of CD105 in pCCA is as yet unclear, although its over-expression in other solid cancers has been shown to confer poorer prognosis [9, 10]. A further consideration with traditional systemic cancer therapies relates to their low therapeutic efficacy and the frequent occurrence of ‘off-target’ or side effects [11]. The development of delivery systems directed against specific molecular targets in tumour tissues provides an attractive approach to mitigate these limitations of standard systemic treatments. One such platform incorporates the use of microbubbles (MBs), which are inert 1–4 μm gas-laden shells that can be functionalised by the attachment of therapeutic ligands to target endothelial receptors. Exposure of MBs to destructive ultrasound (US) promotes their lysis and a subsequent release of MB payloads into tissues in the insonated region [12]. Additionally, by virtue of their gaseous core and ability to be retained within the circulation upon systemic administration, MBs behave as intravascular contrast agents upon diagnostic US application. Thus MBs can be rendered concurrent diagnostic and therapeutic (theragnostic) abilities [13].

In this report, we analyse the expression of CD105 in pCCA tissues along with its prognostic impact. In addition, the ability of a targeted MB platform against CD105 to localise to endothelial cells is analysed using in vitro studies and in vivo CCA models.

## Materials and methods

### CD105 immunohistochemistry of archival human CCA tissues

Approval to source and study archival pCCA tissue samples and relevant patient data was obtained from the Leeds (East) National Research Ethics Service Committee (REC 06/Q1206/136). Consecutive patients who underwent resection of pCCA with curative intent at our institution from January 2000 to September 2010 were identified from a clinical database maintained within the Department of Hepatobiliary Surgery at St James’ University Hospital, Leeds. Representative archival formalin fixed paraffin embedded pCCA tissue blocks from these patients were evaluated by consensus review of corresponding Haematoxylin/Eosin stained slides. Two consecutive 5 μm sections (one for each primary antibody examined) were subsequently obtained from all blocks. All sections underwent serial dewaxing and hydration in successive steps of immersion in Xylene, ethanol and water. Antigen retrieval was performed as necessary prior to endogenous peroxidase quenching with 0.3% *v*/v hydrogen peroxide for 10 min. Primary antibodies were then diluted using antibody diluent reagent solution (Invitrogen, CA, USA) to desired concentrations before application to sections. The latter were then washed in Tris-buffered saline (TBS) with 10% Tween-20, followed by TBS alone. These wash cycles were similarly repeated following subsequent application of species-specific horseradish peroxidase conjugated secondary antibodies for 30 min. Next, incubation was done with DAB+ Substrate Chromogen System (Dako, CA, USA) before slides were counterstained in Haematoxylin, ‘blued’ in Scott’s tap water and dehydrated through immersions in ethanol and Xylene. Permanent mounting under a glass coverslip was done using DPX (Sigma, UK). In order to confirm specificity of staining, ‘no-primary’ antibody slides were included in all runs.

#### Antibodies

The pan endothelial marker CD31 was utilised as reference marker against which the relative expression of CD105 was assessed. The decision to utilize CD31 was based on its comparable profile within normal liver tissue to similar markers such as CD34 [14], as well as prior reports to its validity as a prognostic marker in hilar cholangiocarcinoma [15]. A mouse monoclonal anti-CD31 antibody (Dako, Glostrup, Denmark; Clone JC70A, M0823) was applied at 1/40 dilution and incubation done overnight at 4 °C. For CD105 staining, initial antigen retrieval of sections was done using Proteinase-K enzyme (100 μl per 50 ml phosphate buffered saline, PBS) for 25 min in a water bath at 37 °C. A mouse monoclonal anti-CD105 antibody (Dako, Glostrup, Denmark; Clone SN6H) was applied at 1/20 dilution for 1 h at room temperature.

#### Quantification of endothelial staining

Endothelial staining was assessed using the ‘Hot Spot’ method [16] yielding a Microvessel Density (MVD) score for each section. Hot spots were initially identified by consensus between two scorers using CD31 stained sections, after which MVD scores were calculated independently by both scorers for this antibody, followed by scoring of corresponding spots in adjacent CD105 sections. At the time of assessment, both scorers were blinded to patient variables.

#### Clinical data

Demographic parameters, histopathological data, and follow-up information were collated for all included patients. Tumours were staged in accordance with the 7th edition of the American Joint Committee on Cancer (AJCC) guidelines on perihilar bile duct cancer [17].

### CD105 immunofluorescence assay

To assess the feasibility of targeting CD105 in murine in vitro and in vivo models, immunofluorescence (IF) studies were first conducted using a monoclonal rat anti-mouse antibody directed against CD105 (BD Pharmingen, CA, USA) in the murine endothelial cell line SVEN-1 RAS (SVR) (ATCC, Middlesex, UK). Briefly, cells were grown to confluence on glass cover slips within a 6-well plate (Sigma Aldrich, MO, USA) in RPMI-1640 cell culture medium (Thermo Fisher Scientific, MA, USA). Next, the cells were fixed with 4% *v*/v paraformaldehyde for 10 min at room temperature and subjected to 2 cycles of PBS washes. The CD105 antibody was optimised for use at a concentration of 1/50 in antibody diluent reagent solution (Invitrogen, CA, USA) and added to the cells for 1 h at room temperature. The cells were subsequently washed twice in TBS with 10% Tween-20, followed by TBS alone. A donkey anti-rat Alexa Fluor-594 conjugated secondary antibody (Invitrogen, IL, USA) was then applied at 1/300 dilution (in PBS) to the cells at room temperature in the dark for 1 h. Following a further session of washes, coverslips were mounted onto the slides using Prolong® Gold antifade reagent with 4′,6-diamidino-2-phenylindole (DAPI) (Invitrogen, OR, USA) after which the slides were allowed to dry in the dark overnight. A Zeiss Axioimager.Z1 apotome fluorescent microscope (Carl Zeiss, Thornwood, NY, USA) was used to image the cells at 63x magnification through a Texas Red filter using the AxioVision software package.

### CD105-targeted microbubble preparation

Micromarker™ (Vevo Visualsonics Target-ready contrast agent) microbubbles (MBs; Bracco, Geneva, Switzerland) were reconstituted as per manufacturer’s instructions. Briefly, lyophilized MBs were rehydrated with 700 μl PBS by injection and then agitated. This process generated lipid-based PEGylated shells filled with perfluorobutane and nitrogen gases from the vial headspace. A rat anti-mouse biotinylated CD105 antibody (eBiosciences, CA, USA) and its IgG2a isotype control were each added to separate MB solutions in successive concentrations of 0.05 and 0.1 μg per 10^7^ MBs, respectively. Following gentle agitation for a minute, the mixture was left for 15 min at room temperature prior to use.

#### In vitro microbubble flow assay

To simulate shear stress conditions of capillary blood flow in an in vitro model and thus to determine the targeting capabilities of CD105-coated microbubbles in such an environment, flow assays were set up as described previously [18]. Briefly, 30 μl of 3x10^5^ SVR cells/ml were each plated into 6 channels of a μ-Slide VI^0.4^ (Ibidi, Martinsried, Germany) and incubated at 37 °C in 5% CO_2_ for 48 h until cells reached approximately 80–90% confluence. To allow the cells to grow on the upper surface of the channels, slides were stored in an inverted position during this period. The flow assay assembly consisted of a motorised syringe driver pump (World Precision Instruments, FL, USA) with adjustable flow rate, net volume and syringe diameter functions. Following 3 initial PBS flush cycles to clear away culture media and loose cells, the driver delivered 10^7^ MBs (suspended in PBS) at a rate of 0.2 ml min^−1^ for 5 min via a flexible connector tubing connected into the reservoir at one end of the Ibidi chip channel. Fluid, once traversed the channel, was collected into a reservoir at the opposite end of the chip and continued into a waste pot via a second segment of connector tubing. A further cycle of PBS wash was delivered before analysis of the flow channels by inverted light microscopy (40x magnification) for MB binding. Using a digital camera in-line with the optics of the microscope, 5 images were taken of different fields within each flow channel. MBs were identified in these images as double layered refractile rounded objects. The number of MBs bound to (i.e., in contact with) SVR cells was counted in each image, along with the total number of SVR cells in that field. This process was repeated for all 5 images and the degree of binding expressed as a ratio of bound MBs to total cells.

#### In vivo microbubble binding assays

##### Mouse models

The extra-hepatic CCA cell lines TFK-1 and EGI-1 (DMSZ, Germany) were utilised to create subcutaneous xenografts in female Balb/c (congenitally athymic) nude mice (Charles River Laboratories, MA, USA). The median (range) mouse age was 7 (6–9) weeks. All mice were pathogen free (SPF) and maintained in a dedicated facility ensuring high standard health conditions. They were housed communally in individually vented cages with food and water provided ad libitum, as well as alternating light-dark cycles to simulate diurnal patterns. All interventions were approved by the UK Home Office and carried out as per the Animals (Scientific Procedures) Act 1986 regulations. Following the application of topical anaesthesia, the right flank regions of the mice were inoculated with 1 × 10^7^ CCA cells suspended in 100 μl PBS. Regular calliper measurements of tumour dimensions were subsequently made to verify xenograft growth. Experiments were conducted approximately 4–6 weeks later.

##### MB injection and high frequency ultrasound (HFUS) assessment

The HFUS methodology employed was similar to that previously described by Willmann et al. [19]. Briefly, under inhaled Isoflurane anaesthesia administered by nose cone at 2 L/min, mice were fastened to an electroconductive board that allowed real time monitoring of heart rate and temperature. This assembly was placed under a warming light, after which a 40-MHz linear transducer HFUS probe (RMV-704; VisualSonics) was placed over the xenograft and real time imaging was performed using the Vevo 770 system (Fujifilm VisualSonics). Two-dimensional US mode was used to identify the centre (largest diameter) of the xenograft after which the probe was fixed into position on the assembly. The tail vein of the mouse was identified for intravenous injection. Using a tail vein catheter, initially 50 μl (equivalent to 10^8^) isotype control MBs was injected at a rate of 0.6 ml/min using a motorised syringe driver. Four minutes were given to allow injected MBs to bind to xenograft vasculature and readings were saved. Subsequently, a destructive US pulse (10 MHz) was administered through the fixed probe and readings obtained again. This process was repeated for CD105 MBs after a lapse of 10–20 min, to allow isotype control MBs to be eliminated from the circulation.

In the post-processing phase, the VisualSonics software was utilised to generate a 3-dimensional image and volume of the tumour. This was performed by outlining the xenograft border using the software every 10–20 frames. Next, the final 100 frames in sequence from the experiment (for each antibody run) was taken as a baseline to calculate signal amount from bound and flowing MBs (pre-destruction pulse) and that from flowing MBs alone (post-destruction pulse). The difference between the reference-subtracted mean amplitude of the latter and the former gave the subtracted molecular signal (i.e., signal due to bound MBs alone), indicative of the amount of CD105 receptors in the scanned vasculature. If the subtracted molecular signal was higher with CD105 MBs in comparison to that of isotype control MBs, specific binding was deemed as having occurred.

### CD105 immunohistochemistry of xenograft sections

Following in vivo experiments with CD105 tagged microbubbles, animals were sacrificed and CCA xenografts harvested and paraffin embedded prior to microtome sectioning as described previously. Five-micron sections were dewaxed and hydrated as detailed previously. No antigen retrieval was required. Endogenous peroxidase activity was quenched and an Avidin-Biotin block done using a blocking kit (Vector, Burlingame, CA, USA). A Casein block (Vector, Burlingame, diluted in Antibody diluent reagent solution) was performed by incubation of the sections for 20 min to reduce background staining further. A goat polyclonal anti-mouse CD105 antibody (AF 1320, R&D Systems, Minneapolis, MN, USA) was used at 1/500 dilution for 1 h at room temperature. A polyclonal rabbit anti-goat biotinylated immunoglobulin (Dako, Carpinteria, CA, USA) was used at 1/200 dilution for 30 min for secondary incubation. A Vectastain® Elite® ABC kit (Vector, Burlingame, CA) was then applied to the sections before the addition of DAB. The sections were finally dehydrated through ethanol and xylene before coverslips were mounted and slides dried overnight.

### Statistical methods

The statistical software package SPSS version 22 (IBM, Armonk, NY, USA) was used for data analysis, with a *p* value < 0.05 used to denote statistical significance. Categorical variables were analysed using the Fisher’s exact test, whereas the Mann-Whitney U test was used for comparisons between continuous data. Correlations between continuous variables were assessed with the Spearman test, and the Wilcoxon signed rank test was used for matched tissue comparisons. Using an online tool (http://molpath.charite.de/cutoff) [20], MVD scores and other continuous variables were dichotomised at the best cut-off for survival analysis. Survival curves were generated using the Kaplan-Meier method and differences between proposed predictive factors assessed using the log rank test. Censoring of data towards calculation of survival statistics was done for all cases of peri-operative death, patients who died later due to non-CCA related causes/lost to follow up and in those alive at the end of 5 years. Factors found to be significantly associated with mortality upon univariate analysis were entered into a Cox regression model to ascertain independent determinants of survival.

## Results

### High CD105 MVD is associated with poor prognosis in pCCA

**Fig. 1:**
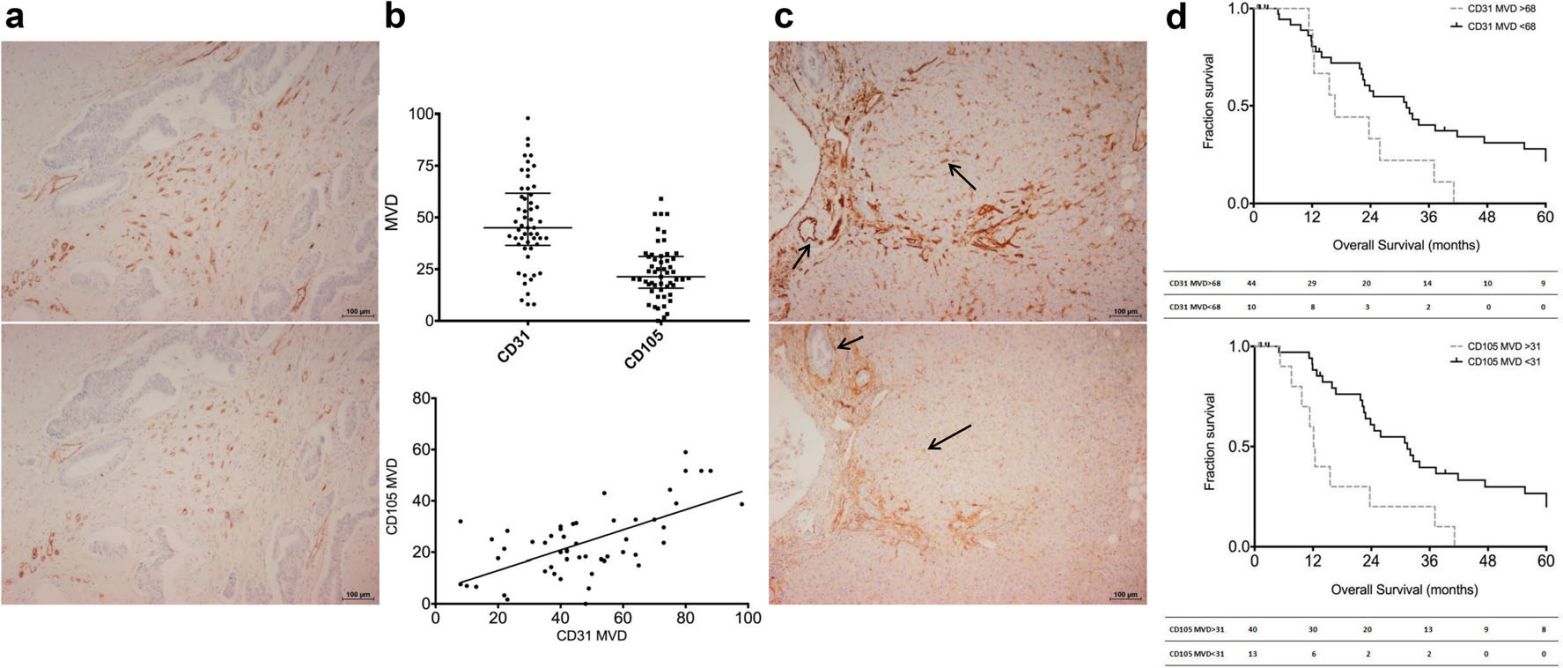
Tumoural CD105 expression correlates to that of the pan-endothelial marker CD31 in CCA and is associated with poorer disease prognosis, but is also present in normal liver parenchyma. (**a**). Consecutive tumour sections showing CD31 (top) and CD105 (bottom) expression patterns. (**b**) MVD expression profiles of CD31 and CD105 in all examined tumour sections (top), with fair correlation observed between the two markers (bottom; Spearman r = 0.51, *p* < 0.0001) (**c**) Positivity is observed in portal tracts (short arrows) and liver sinusoidal cells (long arrows) and for both CD31 (top) and CD105 (bottom). (**d**) Kaplan-Meier curves highlighting a significant difference in overall survival between patient groups at MVD cut-offs for both endothelial markers as denoted (top CD31; *p *= 0.042, and bottom CD105; *p *= 0.002, log-rank tests). Tables below graphs indicate numbers at risk at corresponding time points

A total of fifty-four patients underwent resection of pCCA during the study period. Baseline clinicopathological patient characteristics are summarised in Table 1. The median (inter-quartile range [IQR]) survival of the entire cohort of patients was 22.3 (10.8–39.7) months, whilst the group had a median (IQR) disease-free survival (DFS) of 13.8 (4.8–33.4) months. Overall median patient follow up was 22 (range 1–160) months. The majority of tumour specimens showed positivity for CD31 and CD105 to varying degrees (Fig. 1a). The median (IQR) CD31 MVD was 45.17 (36.2–62) whilst for CD105 the corresponding value was 21.3 (15.8–31.1), and the correlation between the MVD scores for both markers was strong (Fig. 1B). Normal liver sinusoidal tissues showed consistent expression of the markers (Fig. 1c).

**Table 1:** Baseline patient characteristics

**Variable**	**n** (total = 54)	**%**
Median age (range) in years	57.6 (33–81)	
Gender		
Male	31	57.4
Female	23	42.6
Surgical procedure		
Left trisectionectomy (Segments 2,3,4,5,8,±1) & bile duct excision	18	33
Left hemihepatectomy (Segments 2,3,4,±1) & bile duct excision	7	13
Right trisectionectomy (Segments 4,5,6,7,8,±1) & bile duct excision	19	35
Right hemihepatectomy (Segments 5,6,7,8,±1) & bile duct excision	7	13
Orthotopic Liver Transplant	2	4
Bile duct excision alone	1	2
Median Tumour size (range) in mm	30 (10–75)	
Tumour differentiation (grade)		
Well (G1)	27	50
Moderate (G2)	19	35.2
Poor (G3)	8	14.8
Microscopic vascular invasion	33	61.1
Perineural infiltration	48	88.9
Tumour stage		
T1	2	3.7
T2	29	53.7
T3	19	35.2
T4	4	7.4
Nodal stage		
N0	25	46.3
N1	21	38.9
N2	8	14.8
AJCC Stage (7th edition)		
I	0	0
II	15	27.8
IIIA	8	14.8
IIIB	21	38.9
IVA	2	3.7
IVB	8	14.8
Resection margin		
R0 (clear)	21	38.9
R1 (involved)	33	61.1
Local recurrence	5	9.3

The results of univariate analysis of endothelial marker expression against clinicopathological patient variables are shown in Table 2. Lower median CD31 MVD scores were associated with increased rates of local recurrence (i.e., within the surgical bed), whereas CD105 MVD scores bore no significant statistical association with any of the analysed variables. As elaborated in the materials and methods section, MVD scores for CD31 and CD105 were dichotomised at an optimal value for the purpose of survival analysis. This cut-off value was determined separately for each marker so as to generate the maximal statistical significance on log-rank (univariate) analysis. Kaplan-Meier survival curves for CD31 and CD105 at these MVD cut-offs (68 and 31, respectively) revealed that higher expression levels of both these endothelial markers showed a significant association with poorer OS (Fig. 1d). The full results of survival analyses for the analysed variables are shown in Table 3. The median OS in patients with CD105 MVD > 31 was 12 months versus 31 months in the group with CD105 MVD < 31. Neither CD31 nor CD105 MVD was significantly associated with DFS. Other factors that showed statistical significance included primary tumour size > 48 mm (for both poorer OS and DFS) and resection margin positivity (for worse DFS, but not OS). Factors significant by univariate survival analysis were entered into a multivariate (Cox proportional hazards) regression model. This revealed that CD105 MVD > 31 and primary tumour size > 48 mm were independently associated with poorer OS in this cohort of pCCA patients.

**Table 2:** Comparison of MVD scores of CD31 and CD105 against clinicopathological patient variables

**Variable**		**n**	**%**	**CD31 MVD [median (IQR)]**	***p* value**	**CD 105 MVD [median (IQR)]**	***p* value**
Age (years) at diagnosis	< 58	27	50	44 (31–59)	0.482	20 (14–30)	0.203
	> 58	27	50	46 (40–64)		24 (17–33)	
Gender	Male	31	57	42 (23–55)	0.062	21 (17–31)	0.871
	Female	23	43	54 (40–64)		22 (14–31)	
Tumour size (mm)	< 30	24	46	43 (35–59)	0.912	21 (17–31)	0.955
	> 30	28	54	45 (37–64)		24 (14–32)	
Histological grade	Grade 1	27	50	53 (40–65)	0.050	21 (17–30)	0.676
	Grade 2/3	27	50	41 (23–54)		22 (9–32)	
Microscopic vascular invasion	Yes	33	65	44 (34–67)	0.454	24 (16–32)	0.664
	No	18	35	43 (35–54)		21 (18–28)	
Nodal metastases	Yes	29	54	48 (38–69)	0.138	19 (16–32)	0.391
	No	25	46	41 (29–58)		24 (18–31)	
T Stage (AJCC 7th ed)	T1/T2	31	57	45 (23–64)	0.687	21 (17–32)	0.857
	T3/T4	23	43	45 (40–61)		23 (14–30)	
Resection Margins	R0 (negative)	21	39	50 (41–62)	0.245	22(17–32)	0.797
	R1 (positive)	33	61	41 (35–63)		21 (14–31)	
Local recurrence	Yes	5	9	35 (14–39)	0.008	25 (15–28)	0.825
	No	49	91	48 (39–64)		20 (15–32)	
Metastasis	Yes	25	46	42 (22–63)	0.212	24 (18–29)	0.762
	No	29	54	48 (40–62)		20 (12–33)	
Progressive disease	Yes	27	50	42 (23–61)	0.100	24 (18–30)	0.510
	No	27	50	49 (40–64)		20 (11–34)	

Mann-Whitney U tests used for continuous variables and Fisher’s exact test for dichotomous data. Local recurrence defined as recurrent disease in surgical bed. Progressive disease indicating local recurrence and/or metastasis (MVD: Micro-vessel density, IQR: Inter-quartile range)

**Table 3:** Univariate (Log -rank tests) and multivariate (Cox Regression) analysis of factors predictive of survival

			Univariate analysis						Multivariate analysis			
			OS			DFS			OS		DFS	
Variable		n	Median survival in months	HR (95% CI)	*p* value	Median survival in months	HR (95% CI)	*p* value	HR (95% CI)	*p* value	HR (95% CI)	*p* value
CD31 MVD	< 68	44	31.4	2.2 (1.0–4.9)	0.042	31.2	1.8 (0.7–4.5)	0.212	0.2 (0.03–1.5)	0.132		
	> 68	10	16.7			18.3						
CD105 MVD	< 31	13	31.4	3.2 (1.5–6.8)	0.002	23.1	1.7 (0.6–4.6)	0.292	12.5 (1.9–79.9)	0.007		
	> 31	40	12.2			13.6						
Nodal metastasis	Yes	29	21.7		0.085	17.3		0.634				
	No	25	32.6			31.2						
Tumour size	< 48 mm	40	32.0	4.1 (1.6–10.6)	0.002	23.2	3.3 (1.2–9.5)	0.019	5.0 (1.9–13.4)	0.001	2.6 (0.9–7.7)	0.071
	> 48 mm	12	12.7			11.2						
Tumour stage	T1/T2	31	31.4		0.277	31.2		0.530				
	T3/T4	23	23.8			18.7						
Tumour grade	Grade 1	27	25.9		0.220	23.2		0.556				
	Grade 2/3	27	24.6			18.7						
Vascular invasion	Yes	33	24.6		0.177	18.3		0.650				
	No	18	32.0			32.9						
Resection margin	Positive	33	23.7		0.203	18.3		0.032			2.1 (0.9–5.2)	0.080
	Negative	21	37.0			51.2						
AJCC stage	II	15	37.3		0.116	32.9		0.504				
	III/IV	39	22.8			18.3						
Local Recurrence	Yes	5	33.9		0.568			N/A				
	No	49	23.8									
Metastasis	Yes	25	24.6		0.154			N/A				
	No	29	33.9									

Factors significant on univariate analysis entered into the Cox Regression model. OS: overall survival, DFS: disease-free survival, HR: Hazard ratio, CI: Confidence interval, MVD: micro-vessel density

### CD105-MBs bind mouse endothelial cells under conditions of simulated capillary flow

**Fig. 2:**
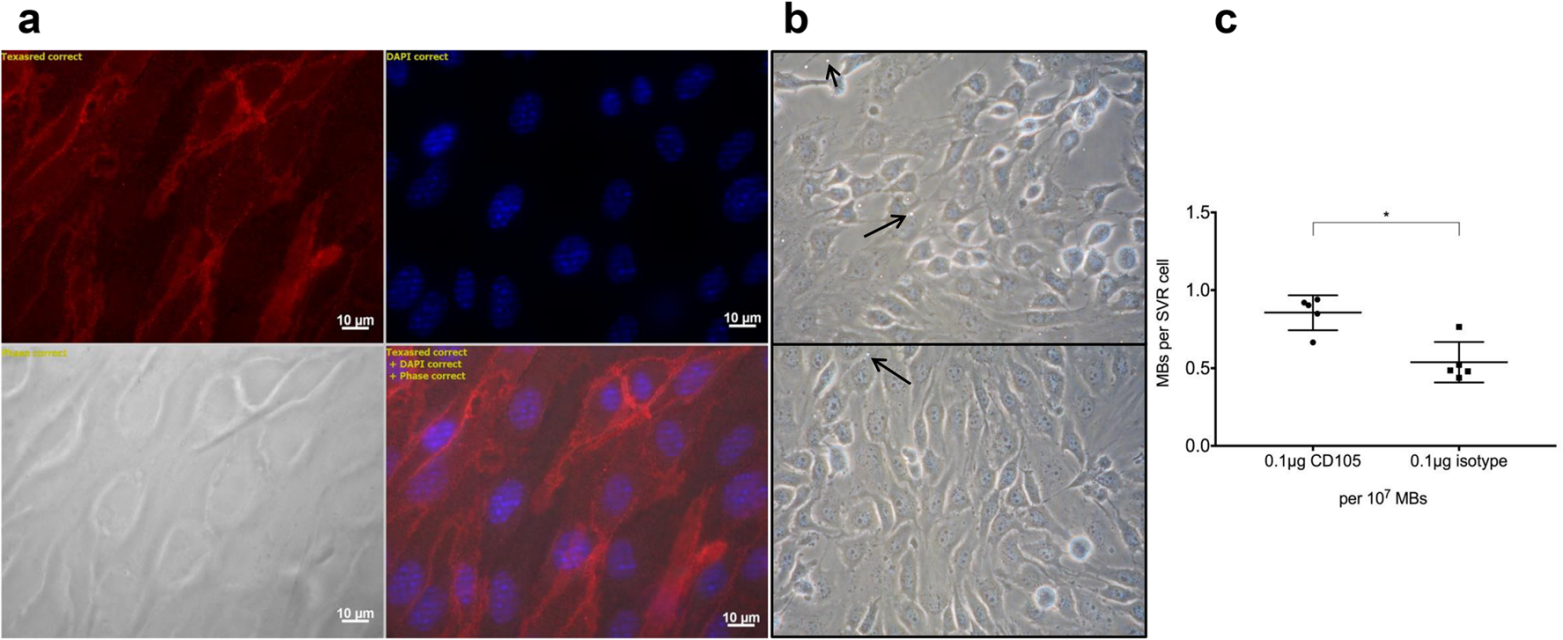
CD105 is expressed on the surface of murine SVR endothelial cells whereas CD105 functionalized MBs bind SVR cells under conditions simulating capillary flow. (**a**) Immunofluorescence images showing surface expression of CD105 on SVR cells (top left CD105 expression, top right DAPI nuclear staining, bottom right composite image and bottom left phase contrast image). (**b**) Images from flow assay experiments using CD105-MBs (top) and isotype control-MBs (bottom). Long arrows denote bound MBs (in contact with cellular membrane), whereas unbound MBs are free standing (short arrow). (**c**) Graphical representation of flow assay results showing significantly higher binding of SVR cells to CD105-functionalised MBs in comparison to isotype-control-MBs, at an antibody concentration of 0.1 μg/ml (*p = 0.016) Data expressed as median with interquartile range (n = 5 runs; Mann-Whitney U test)

Using immunofluorescence on fixed sections of murine SVR endothelial cells, we observed a strong CD105 surface expression (Fig. 2a). Further to this, in vitro flow assays simulating capillary blood flow were established, and revealed significantly increased binding of CD105-MBs to SVR cells in comparison to isotype control, at an antibody concentration of 0.1 μg per 10^7^ MBs (Fig. 2b and c).

### Ultrasound assessment of a heterotopic subcutaneous CCA model with CD105-MBs reveals xenografts vascularized with CD105 positive endothelium

**Fig. 3:**
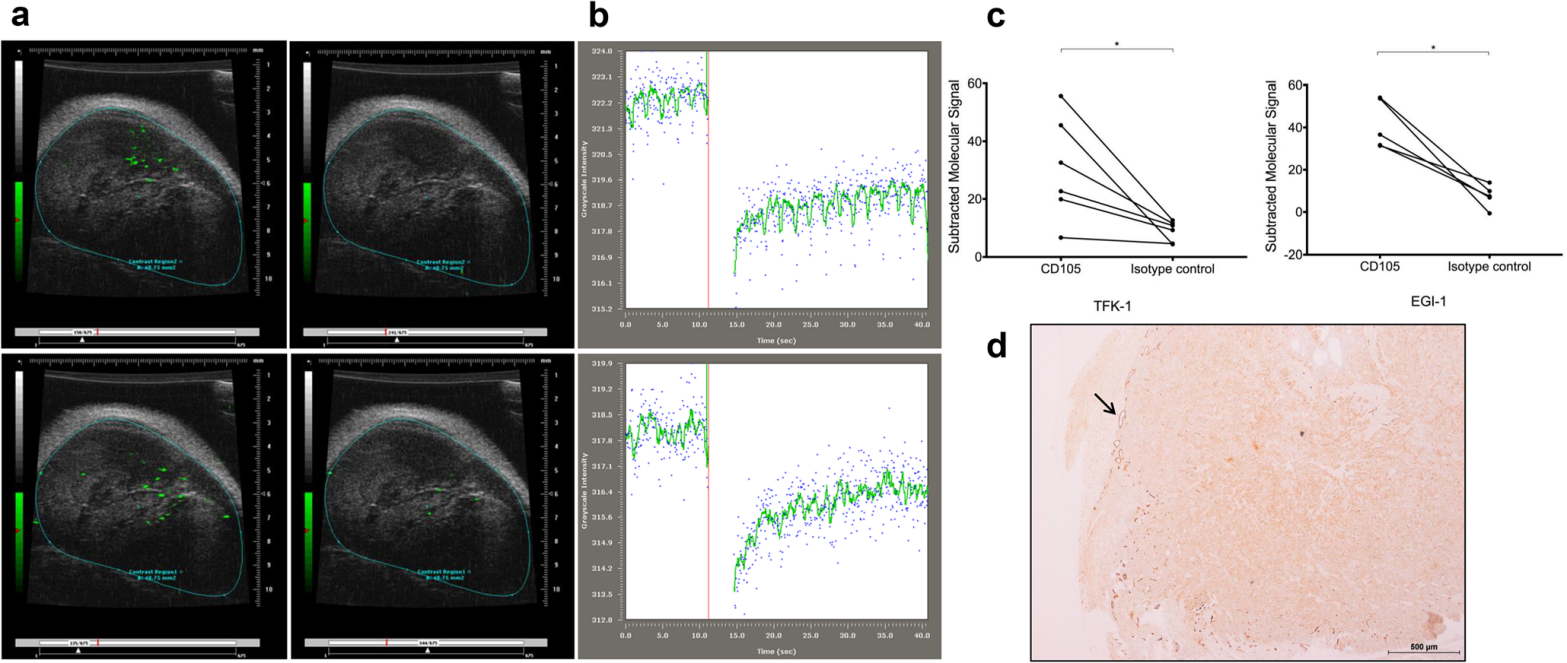
In vivo US imaging of cholangiocarcinoma xenografts with CD105-MBs reveals significant binding totumour endothelium. (**a**) US imaging of a TFK-1 xenograft following administration of MBs bound to CD105 antibodies (green signal), indicative of both flowing and bound MBs (top left), and following application of destructive US pulse showing only flowing MBs (top right). Similarly shown are signals observed with isotype-control antibody bound MBs before (bottom left) and after (bottom right) destructive US pulse. (**b**) Images demonstrating the difference in molecular signal intensity prior to and after (left and right respectively of vertical red line) application of destructive US pulse, for CD105-MBs (top) and isotype-control MBs (bottom). (**c**) A significant difference in subtracted molecular signal (representative of the degree of binding to tumour endothelium) is observed between CD105-MBs and its isotype control when xenografts are subjected to a destructive US pulse (TFK-1 *p* = 0.028 [n = 6] and EGI-1 *p* = 0.043 [n = 4]; Wilcoxon sign rank tests). (**d**) Xenograft section showing abundance of CD105 vessels (arrow) at tumour periphery

The extrahepatic cholangiocarcinoma cell lines TFK-1 and EGI-1 were used to establish subcutaneous xenografts in Balb/c nude mice (*n* = 6 and *n* = 4, respectively), after which targeted microbubble binding assays were conducted as shown in Fig. 3a and b. Following injection of CD105-MBs, real-time high-frequency US assessment of the xenografts before and after application of a destructive 10 MHz US pulse revealed significant binding of CD105-MBs compared to isotype control in both tumour types (Fig. 3c). CD105 immunohistochemistry of xenograft sections revealed abundant peri-tumoural endothelial cells positive for the marker, giving credence to the in vivo US findings (Fig. 3d).

## Discussion

Neovascularization enhances both local aggressiveness and metastatic potential in tumour tissue by increasing nutrient and oxygen delivery to cancer cells. The process of angiogenesis in this environment stems from an interplay of various factors secreted by tumour cells and cancer-associated stromal cells, and is mediated through several signalling pathways [21]. Here we demonstrate that CD105 expression is an independent adverse prognostic factor in pCCA whilst also possessing properties that render it suitable as a ligand for MB delivery to CCA tissue in vivo. CD31 was utilised primarily as a reference endothelial marker in this study although its impact in CCA prognosis has also been analysed previously. In a series of 60 pCCA patients who underwent resection, tumours with high CD31 MVD (based on a cut-off value of 20) were found to be significantly linked to higher risk of nodal involvement and local recurrence, whilst upon Cox regression analysis high MVD was found to be an independent predictor of adverse OS [15]. In contrast, higher expression of CD31 was significantly associated with poorer OS on univariate analysis in our cohort, although no prognostic impact was seen using Cox regression analysis.

When considering CD105, there are currently no published series that delve into the expression of this marker in CCA tissues. Our work scrutinizes the expression and prognostic impact of CD105 MVD in pCCA, and although this bore no significant statistical association with any patient features, we were able to show that a MVD > 31 was associated with a significantly poorer OS. In addition to the prognostic effect of CD105 MVD in our cohort, an equally pertinent observation that we made was the consistent expression of CD105 in hepatic sinusoidal endothelium. Similar findings have been noted by others, with CD105 expression observed mostly in the outflow region of the sinusoids, adjacent to the terminal hepatic vein [22, 23]. Previous studies have also documented expression of CD105 in both tumour (metastatic and HCC) and non-tumour bearing liver parenchyma [24–26] and put forth the view that in peri-tumoural areas its presence is perhaps indicative of a field change that predisposes to tumour progression [24]. Nonetheless, our findings bear relevance for the development of a targeted particles directed against CD105, as off-target effects on hepatocytes would not be surprising under these circumstances. However, the use of focused high-frequency US to increase the specificity of payload targeting by MBs can in theory improve the degree of such lateral effects. The expression profile of CD105 in non-‘hot spot’ areas of CCA tissues (e.g. peri-tumoural regions and in tumour lymphatics) is of interest in relation to its potential prognostic impact. Nonetheless, in this report we focused purely on hot-spot assessment since highly vascularized areas are arguably more relevant to the delivery of systemically administered MB platforms. Although targeting angiogenesis in CCA is an attractive therapeutic option, multiple recent phase-II clinical trials of angiogenic inhibitors in advanced CCA failed to lead to significant improvements in patient outcomes [27]. The main focus of these studies has revolved around blockade of the VEGF axis, and the lack of positive results suggests the importance of alternative angiogenic pathways in the pathogenesis of biliary tract cancers. Nonetheless, vascular targeting may not involve inhibition alone. Capillary vascular endothelium is the first physical barrier between the bloodstream and the tumour interstitium, and this barrier must be crossed in order for therapeutics to exert their actions. Therefore, the concurrent use of tumour endothelium as an anchor point to deliver payloads specifically into tumour masses may improve treatment efficacy and minimize off-target effects. MBs are well suited to this purpose and can be functionalised through the addition of ligands to its surface that enable them to bind vascular endothelium. Additionally, MBs possess properties that render them susceptible to both the diagnostic and therapeutic effects of ultrasound. The first step towards exploring the feasibility of targeting tumour endothelium in an in vivo model was to verify CD105 expression in murine endothelial cells, as xenografts are supplied by native vessels. Accordingly, our immunofluorescence experiments confirmed CD105 positivity on the surface of murine SVR endothelial cells. Next, we showed that CD105-MBs could bind SVR cells appreciably more than isotype control MBs under conditions of simulated capillary flow. Finally, our murine in vivo MB experiments revealed that these intravascular agents, when functionalised with surface CD105 ligands, were able to bind CCA xenograft endothelium at significantly higher levels than that seen with MBs with isotype control. The use of CD105-conjugated MBs has been reported before, where MBs were functionalised with biotinylated CD105 by incorporation of avidin into the MB shell. Using tagged fluorophores and in conditions of stasis (rather than flow), it was demonstrated that such targeted MBs bound to endothelial cells significantly better than control MBs [28]. The same group showed that MBs conjugated with CD105 and VEGFR2 ligands were able to bind pancreatic cancer xenograft vasculature significantly better than control MBs, using contrast US imaging in a murine model. Mice treated with Gemcitabine showed a significant reduction in vascularity compared to control as demonstrated with CD105-MB [29]. More recent work has highlighted successful transfection of breast cancer xenografts in mice with the endostatin gene using CD105 conjugated MBs by means of US-directed MB destruction. Xenografts successfully transfected in this fashion showed significantly higher levels of endostatin expression compared to control, along with significant reductions in tumour volumes [30].

An inherent limitation of this study is the use of heterotopic CCA models for assessment of MB binding. Murine subcutaneous xenograft cancer models, though a recognised platform for the assessment of cancer targeting, suffer the detriment of not accounting for the influence of a natural tumour microenvironment, on which orthotopic syngeneic cancer models shed more light [31]. The engrafting of human CCA xenografts within the liver is however technically challenging, although our group has had recent success in this respect utilizing ultrasound-guided injection into the liver [32]. In addition, it would have been ideal to conduct our in vivo experiments with a hilar CCA cell line, but the use of extra-hepatic CCA cell lines was favoured due to their availability, ease of xenograft establishment and reproducibility. Although heterogeneity does exist between these two topographical forms of CCA, they are nonetheless closely related in behaviour and prognosis.

Through the use of in vitro and in vivo methods, we have shown that CD105-functionalised commercial MBs bind SVR cells significantly better than isotype control MBs under conditions of flow. The degree of off-target delivery to normal liver tissue is, however, unknown and may be a potential Achilles heel of any targeted delivery system that relies on a homing biomarker that is expressed in both tumour and liver sinusoidal endothelium. Nonetheless, by virtue of being able to generate shear stresses on cell membranes under the influence of focused ultrasound (thus increasing cellular permeability via sonoporation), MBs are able to offer enhanced intracellular molecular delivery [18]. Anti-CD105 therapies have already entered the realm of phase II clinical trials in various solid malignancies. The use of an IgG1 chimeric monoclonal antibody (TRC105) in advanced HCC has shown to exhibit an acceptable safety profile, with evidence of anti-tumour activity in combination with Sorafenib [33]. What the future holds for this marker is yet to be fully determined, but our findings provide a basis for further exploration of its potential in biliary cancers.
